# The Quiet Embryo Hypothesis: 20 years on

**DOI:** 10.3389/fphys.2022.899485

**Published:** 2022-05-11

**Authors:** Henry J. Leese, Daniel R. Brison, Roger G. Sturmey

**Affiliations:** ^1^ Centre for Atherothrombosis and Metabolic Disease, Hull York Medical School, University of Hull, Hull, United Kingdom; ^2^ Department of Reproductive Medicine, Old St. Mary’s Hospital, Manchester University NHS Foundation Trust, Manchester, United Kingdom; ^3^ Division of Developmental Biology and Medicine, School of Medical Sciences, Faculty of Biology, St Mary’s Hospital, Medicine and Health, the University of Manchester, Manchester University NHS Foundation Trust, Manchester, United Kingdom

**Keywords:** metabolism, embryo, blastocyst, amino acids, pyruvate

## Abstract

This article revisits the hypothesis, proposed in 2002, that the successful development of oocytes and preimplantation mammalian embryos is associated with a metabolism which is “quiet” rather than “active”, within limits which had yet to be defined. A distinction was drawn between Functional Quietness, Loss of quietness in response to stress and Inter-individual differences in embryo metabolism and here we document applications of the hypothesis to other areas of reproductive biology. In order to encompass the requirement for “limits” and replace the simple distinction between “quiet” and “active”, evidence is presented which led to a re-working of the hypothesis by proposing the existence of an optimal range of metabolic activity, termed a “Goldilocks zone”, within which oocytes and embryos with maximum developmental potential will be located. General and specific mechanisms which may underlie the Goldilocks phenomenon are proposed and the added value that may be derived by expressing data on individual embryos as distributions rather than mean values is emphasised especially in the context of the response of early embryos to stress and to the concept of the Developmental Origins of Health and Disease. The article concludes with a cautionary note that being “quietly efficient” may not always ensure optimal embryo survival.

## Introduction

In 2002, one of us proposed that *preimplantation embryo viability was associated with a* “*quiet*” *rather than* “*active*” *metabolism* (Leese, 2002). This succinct conclusion arose from the culmination of a number of datasets, particularly on the nutrition of single embryos measured non-invasively (e.g., Houghton et al., 2002; Brison et al., 2004). These observations provided information on inter-individual differences, which it was hoped might 1 day deliver a biomarker to enable the selection of embryos most suitable for transfer in clinical IVF. However, before revisiting this evidence and more recent findings, it is useful to consider two further categories of quiet metabolism. These were defined by Leese et al. (2008) as “*Functional Quietness*”, in which metabolic activity changes as part of the embryo’s natural developmental programme, and “*Loss of quietness in response to (environmental) stress”*. Much of the information in these two categories was historical and obtained from experiments on pooled embryos.

Examples of Functional Quietness.

In this part, we use the term “quiet”, or “quieter” to refer to metabolic activity. For example.

• Cleavage stage embryos consume less glucose and oxygen than do blastocysts, and thus are less active metabolically, i.e., “quieter” (Brinster, 1973).
• Pyruvate and oxygen consumption per mouse oocyte increases two- and ninefold, respectively, between the primary and pre-ovulatory stages (Harris et al., 2009).
• Amino acid uptake and incorporation into protein by mouse embryos are low and relatively constant until the early blastocyst (day 3) stage of development when they increase 3- to 9-fold (Epstein and Smith, 1973)
• Cells of the inner cell mass are quieter than those of trophectoderm (Houghton, 2006).

Examples of loss of quietness in response to environmental stress.

• Protein synthesis (Baumann et al., 2007) and amino acid depletion (Sturmey et al., 2010) is higher for in vitro-versus in vivo-produced cattle embryos
• Mouse blastocysts cultured singly from the 2-cell stage in medium KSOM + amino acids showed a ∼3-fold increase in the incidence of cell death, predominantly in the ICM, relative to blastocysts formed *in vivo* (Brison & Schultz, 1997). Rubio Pomar et al., 2005 later confirmed that the extent of apoptosis is much greater for *in vitro* -produced embryos than those conceived *in vivo* in a variety of farm animal species (Rubio Pomar et al., 2005).
• Exposure to elevated oxygen may increase Reactive Oxygen Species production and ensuing oxidative damage in preimplantation embryos (Sturmey et al., 2008).
• Increased glucose consumption and conversion to lactate is a well-known feature of stress in somatic cells, discussed in the context of the early embryo by Leese et al., (1998).

For further examples and a discussion of each of these categories, see Leese et al. (2008). It remains a major goal of IVF and related technologies to devise protocols which counteract such stressors and enable embryos to retain their *in vivo* phenotype but this requires significant endeavour, not least in the human, where in vivo-derived embryos are not available for research purposes.

### Inter-Individual Differences in Embryo Metabolism

A critical feature of studying early mammalian embryos is that, by definition, it is the study of individual entities. Each single embryo is as unique as each individual animal or person, with an exclusive genotype manifesting as a distinctive phenotype. When conducting experiments on early embryos, researchers often attempt to identify generalisable patterns, through replication; this may be by studying replicate embryos from individual parentage (i.e., “sibling” embryos) or from a more diverse spread of parents, such as in-bred animal models or gametes and embryos from outbred individuals. In the case of the human, embryos are frequently siblings since most cycles will provide more than one embryo which could be used for research purposes, especially when they are frozen/thawed. While generalisable patterns are crucial for our understanding of early development, and a topic to which we return later in the essay, it is important to reflect on the power of studying single embryos, which enables distinctive, subtle differences in metabolism to be distinguished. Some examples of the use of this approach are as follows:

• The metabolism, in terms of the turnover (the sum of depletion and appearance) of a mixture of 18 amino acids by single human embryos in culture is lower for those day 2, 3 embryos that reach the blastocyst stage compared with those that arrest (Houghton et al., 2002), predictive of which day 1, 2 embryos achieve a pregnancy following transfer (Brison et al., 2004) and which day 2, 3 cryopreserved human embryos develop to blastocysts in culture (Stokes et al., 2007), as well as differing between uniformly euploid versus aneuploid embryos (Picton et al., 2010).
• Pyruvate consumption by day 2 and day 3 human embryos which subsequently gave a pregnancy was significantly lower compared to embryos which failed to implant (Conaghan et al., 1993).
• Bovine *oocytes* with the capacity to cleave following fertilisation have a lower amino acid turnover than those that arrest (Hemmings et al., 2012).
• Bovine embryos with a low amino acid metabolism are more likely to form blastocysts than those with a high metabolism (Sturmey et al., 2010).

The hypothesis, which became known as the “Quiet Embryo Hypothesis” has generally been received positively and may have been useful in countering the belief which is widespread in IVF that “more is better”. At the time of writing, the 4 main publications on “quiet metabolism” have in total been cited ∼900 times. In most cases, the hypothesis has been used to reinforce or clarify a given experimental approach or interpret a set of findings.

For example, the hypothesis has been invoked to help explain how the endometrium selects embryos of high quality for implantation, rejecting those that emit a “noisy” metabolic signal, characteristic of the high nutrient turnover of arresting embryos, and favouring those that are “quiet” (Macklon and Brosens, 2014; Teklenburg et al., 2010). Measurements of amino acid turnover may also be good candidates for distinguishing high from low embryo quality as sensed by human endometrial stromal cells in patients with a history of recurrent miscarriage (Weimar et al., 2012). Furthermore, maternal obesity during the periconceptional period may compromise the normally quiet metabolism of oocytes and embryos leading to impaired mitochondrial function and the formation of harmful levels of reactive oxygen species (Poston et al., 2011). In addition, elevated levels of mtDNA in the blastocyst may reflect stress that leads to increased energy requirements and reduced developmental potential; consistent with the quiet embryo hypothesis (Fragouli et al., 2015).

Interpretation of the Quiet Embryo Hypothesis has reached beyond human early embryo development. In an experiment on bovine oocytes, those with higher and lower developmental potential after IVF gave different amino acid profiles, which lead the authors to conclude that: “*Overall, poorer quality is related to higher amino acid turnover, in agreement with Leese’s “quiet embryo hypothesis”* (Collado-fernandez et al., 2012). Genes involved in nucleotide metabolism and lipid metabolism are up-regulated after vitrification of bovine embryos, consistent with the hypothesis (Aksu et al., 2012). In a mouse model, the abnormal metabolic response of oocytes and embryos to a high-fat or nutrient-rich diet, as in obesity, has also been reported as being consistent with their physiologically “quiet” metabolism (Purcell and Moley, 2011). Beyond preimplantation development, Burton et al. postulated that early placental development can be seen as a continuation of the “quiet metabolism” in the preimplantation embryo (Burton et al., 2017).

### The Distribution of Individual Values of Metabolic Markers of Embryo Health.

In the original quiet embryo hypothesis, a number of testable propositions arose, one of which stated that *“the most viable preimplantation embryos - - would have the lowest overall metabolism within limits yet to be defined.* The notion of limits or a *“range of nutrient uptakes” within a given distribution most conducive to viability”* was pursued in a follow-up paper on the molecular characteristics that might characterise a physiologically normal embryo (Baumann et al., 2007)*.* For example, it was proposed that viable embryos carried out DNA replication to a high degree of fidelity such that less energy had to be devoted to DNA repair, thus exhibiting a ‘quiet’ metabolic phenotype. Data in support of this proposition were subsequently obtained by Sturmey et al. (2008) who reported that DNA damage was related positively to metabolic activity (amino acid turnover) in bovine, porcine and human blastocysts.

In a further paper, Leese et al. (2007) put forward the idea that ‘*quiet metabolism is not about “one size fits all” but rather, that for any given set of circumstances - - there is an optimal range of embryonic activity consistent with successful developmental progression’* The notion was well-illustrated by data from Lopes et al. (2007) on the oxygen consumption of *in vivo* derived bovine embryos, where values below 0.78 nl/h or above 1.10 nl/h were associated with pregnancy rates of 48 and 25% respectively while embryos with values between the two boundaries all gave a pregnancy.

### Experimental Test of the Existence of an Optimal Range of Metabolic Activity

**FIGURE 1:**
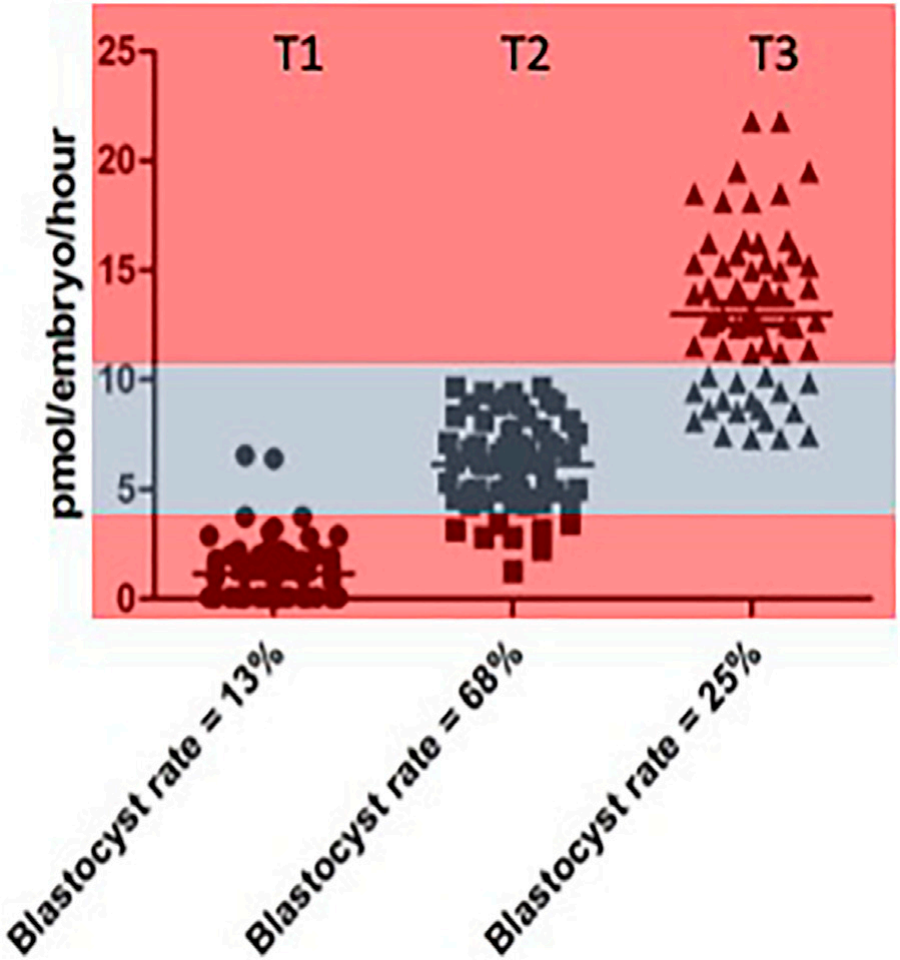
Rate of blastocyst development accourding to the level of pyuvate consumption measured between Days 2 and 3 individual values for pyruvate consumption by bovine embrys assigned prospectivetly to one of three catagories respresting “low” (< 4 pmol per embryo per hour). Data reproduced from Guerif et al (2013) and reproduced under a creative commons licence.

The opportunity to explore the notion of an optimal range of metabolism conducive to successful embryo development rigorously, was eventually realised through experiments carried out by Guerif et al. (2013). This was a prospective study in which the metabolism, in terms of pyruvate consumption, of 30 individual cattle embryos was measured across days 2, 3 of development. On the basis of data obtained, embryos were allocated into one of 3 groups or Tertiles: “low” (T1), “intermediate” (T2) and “high” (T3) pyruvate consumption. The embryos in these groups were then replaced in group culture and allowed to develop for a further 4 days. The experiment was repeated six times—giving 180 embryos in total. The results for pyruvate uptake in the three groups and their respective blastocyst rates are shown in Figure 1.

While the highest rates of blastocyst formation tended to cluster below the median value region of the distribution, it was clear that the quiet hypothesis needed to be modified from binary categories of “quiet” and “active” to encompass the notion of an optimal range or “quiet zone” of metabolic activity within which embryos with maximum developmental potential will be located. In other words, it is not a simple question of “one size fits all”. The considerable overlap between the three categories should also be noted. These issues were discussed in a subsequent paper (Leese et al., 2016) using the concept of a *Goldilocks Zone*, or as it is known in Sweden, *Lagom*, meaning, neither “too much” nor “too little” but “just the right amount” to illustrate the pattern of data distribution.

### Mechanisms Underlying the Goldilocks Distribution

To account for the distribution of individual values, we proposed, an overall, “global” mechanism in terms of energy efficiency and some specific, molecular mechanisms (Leese et al., 2019). The global mechanism implies carrying out a defined action successfully with the minimum *necessary* input of energy (Leese et al., 2019). Thus, the lower limits of the Goldilocks zone will be determined by the threshold value that embryo metabolic activity must reach to satisfy energetic demand for faithful development to proceed, while the upper limit will be set by the physiological scope to increase cellular metabolism, taking into account *the energy parsimony in almost everything they* (living cells) *do* (Johnson, 2013). Beyond this, energetic demand outwith the Goldilocks Zone can obviously be above or below; above the zone indicates an increase in metabolic function to respond to processes beyond the traditional “status quo”; for example, a need to repair cellular damage, while below the zone implies the existence of embryos that are compromised in a manner which precludes attempts to address this by increasing their metabolism. In the case of the developing embryo, it is important to note that such limits are likely to differ for each nutrient or combination of nutrients, the culture medium, developmental stage and the heterogeneity between individual embryos.

Such considerations may help clarify an apparent discrepancy within the literature (e.g., (Ferrick et al., 2020)) in that some researchers have postulated that the best embryos within a given cohort have the highest metabolism, at least in terms of glucose consumption at the blastocyst stage, citing as critical evidence the point that our own studies on the human were all done in a gas phase of 5% C0₂ in air (20% oxygen), which is well-known to act as a source of stress in early embryos. However, our results on “quiet metabolism” in bovine embryos, referenced above, all of which were grown under 5% CO_2_/5% O_2_have always been as pronounced as those for human embryos under 20% oxygen, and included the work above which revealed the Goldilocks zone. In a further paper (Leese et al., 2019) we reported that Goldilocks patterns occur widely within biology and medicine but are by no means universal. Defining the various zones for preimplantation embryo under different conditions can nevertheless, be addressed experimentally as long as individual values are measured.

**FIGURE 2:**
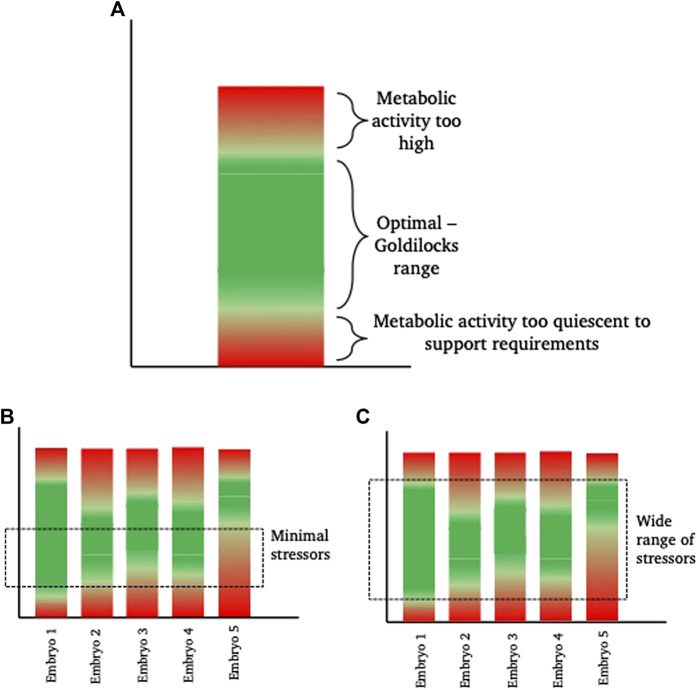
Diagrammatic representation of inter-individual “Goldilocks zone.” **(A)** shows a hypothetical metabolic range for a theoretical embryo. This embryo has a green “optimal” range of metabolism where energetic needs for development are sufficiently high to satisfy demand, but sufficiently low to prevent unintended damage, for example from reactive oxygen species, that are a by-product of elevated metabolism. Within the “green” zone, there is space to increase or decrease metabolism as required (sometimes referred to as metabolic scope), without falling into the damaging ‘red’ zones. **(B,C)** show 5 different theoretical embryos, with inherent metabolic variation. In scenario **(B)**, a minimal exposure to a stressor, such as a low stress environment (for example, culture in optimal conditions with low oxygen tension, optimal nutrient supply and minimal disturbance), keeps more of the embryos within their individual “goldilocks zone” such that they are able to develop. From a selection perspective, embryo 5 is the least viable. In scenario **(C)**, the range of stressors is wide (for example, culture in high oxygen conditions, in poor quality medium and frequent disturbance) meaning that embryos need to respond. Those with the widest inherent “goldilocks zone” (illustrated by Embryo 1) are best able to tolerate the stress and from a selection perspective, Embryo 1 is therefore the most viable embryo.

In relation to this, the range of responses observed in a cohort of embryos is of course dependent on the level of stress encountered. As described earlier, individual embryos are discrete entities and as such, will differ in their ability to respond to, tolerate and recover from, a stressful insult; much like the way that some individuals are able to recover from a given illness faster than others. Thus, there will be a range of responses by individual embryos to an increased stress. This enables embryo selection by selecting those embryos that are able to develop with the minimal observable response to the stress. However, this makes it a practical necessity to identify the embryos responding least favourably to stress for exclusion them from transfer. Conversely, if ART stressors were minimised, variance in gross embryo development might be reduced as a consequence of a narrowing of this range of responses (Figure 2).

### Developmental Origins of Health and Disease

It is well recognised that conditions (especially nutritional) during the periconception period can influence the health of the conceptus and new born, and potentially, the offspring in later life. This phenomenon, which began as the “*Fetal Origins Hypothesis* of Barker (2004) is now known as the *Developmental Origins of Health and Disease (DOHaD)* and is believed to be mediated to a major extent, by epigenetic responses to prevailing conditions, leading to a modification in phenotype that can persist throughout preimplantation and fetal development into childhood and adult health. Increasing evidence from clinical ART research shows a clear link between specific factors in the pre- and peri-implantation embryonic environment (e.g., culture medium composition and use of embryo freezing) and fetal and child growth patterns (Dumoulin et al., 2010; Hann et al., 2018). It is also becoming increasingly clear that the conditions that the embryo is exposed to during the preimplantation stage alters metabolic activity, and the link between metabolism and epigenetic programming in other cell types is widely accepted (e.g., Kaelin and McKnight, 2013). Preimplantation development is particularly susceptible to environmentally induced perturbations leading to impaired future health and for this reason it is critical to try and ensure that the metabolism of the embryo is in the appropriate zone and that the upper and lower limits of its survival are defined.

Finally, we are grateful to a cautionary note from the Ecological Physiologist, Alfred F Bennett, author of a landmark paper on the “Tyranny of the Golden Mean” (Bennett, 1987), who has reminded us that “we must always remember that the driver of natural selection is differential reproduction and that operates on the organismal level. Traits must be harmonized and integrated within an individual and selection operates on that integrated whole. If inefficiency in a process happens to contribute better to organismal function, it may be selected in spite of the inefficiency”. In other words, not everything need be ‘quietly efficient’. There is clearly much still to learn.
